# Comparison of bleeding risk and hypofibrinogenemia-associated risk factors between tigecycline with cefoperazone/sulbactam therapy and other tigecycline-based combination therapies

**DOI:** 10.3389/fphar.2023.1182644

**Published:** 2023-06-07

**Authors:** Lei Zhang, Xinfeng Cai, Fangchen Peng, Shuangshuang Tian, Xinjing Wu, Yun Li, Jinlin Guo

**Affiliations:** ^1^ Department of Pharmacy, Shanxi Provincial People’s Hospital, Taiyuan, China; ^2^ Department of Pharmacy, Shanxi Province Cancer Hospital, Shanxi Hospital Affiliated to Cancer Hospital, Chinese Academy of Medical Sciences, Cancer Hospital Affiliated to Shanxi Medical University, Taiyuan, China; ^3^ Department of Nephrology, Shanxi Provincial People’s Hospital, Taiyuan, China; ^4^ Department of Pharmacy, Yuncheng Central Hospital, Yuncheng, China

**Keywords:** tigecycline, cefoperazone/sulbactam, carbapenems, β-lactam antibiotics, coagulation disorders, hypofibrinogenaemia

## Abstract

**Background:** Tigecycline and cefoperazone/sulbactam can cause coagulation disorders; tigecycline may also lead to hypofibrinogenemia, raising safety concerns. This study aimed to investigate whether tigecycline plus cefoperazone/sulbactam increases the risk of bleeding compared with other tigecycline-based combination therapies and identify risk factors for tigecycline-associated hypofibrinogenemia.

**Methods:** In this multi-method, multicenter, retrospective study, coagulation and other baseline variables were compared using a cohort study, and risk factors for hypofibrinogenemia using a case-control study.

**Results:** The 451 enrolled participants were divided into three group: tigecycline plus cefoperazone/sulbactam (Group A, 193 patients), tigecycline plus carbapenems (Group B, 200 patients) and tigecycline plus β-lactams without N-methylthio-tetrazole (NMTT) side chains (Group C, 58 patients). Activated partial thromboplastin time and prothrombin time were prolonged, and fibrinogen declined for all patients after tigecycline-based medication (all *p* < 0.05). Prothrombin time in Group B was significantly longer than in other groups (*p* < 0.05), but there were no significant differences in bleeding events between the three groups (*p* = 0.845). Age greater than 80 years (OR: 2.85, 95% CI: 1.07–7.60), treatment duration (OR: 1.29, 95% CI: 1.19–1.41), daily dose (OR: 2.6, 95% CI: 1.29–5.25), total bilirubin (OR: 1.01, 95% CI: 1.01–1.02) and basal fibrinogen (OR: 1.32, 95% CI: 1.14–1.63) were independent risk factors of hypofibrinogenemia. The optimal cut-off for treatment course was 6 days for high-dose and 11 days for low-dose.

**Conclusion:** Tigecycline plus cefoperazone/sulbactam did not increase the risk of bleeding compared with tigecycline plus carbapenem, or tigecycline plus β-lactam antibiotics without NMTT-side-chains. Coagulation function should be closely monitored in patients receiving tigecycline treatment.

## Introduction

Carbapenem-resistant Gram-negative organisms are a serious global challenge for physicians, including carbapenem-resistant *Enterobacterales*, carbapenem-resistant *Acinetobacter baumannii* (CRAB) and carbapenem-resistant *Pseudomonas aerugnosa*. CRAB is one of the main causative pathogens of hospital-acquired infections in China. Due to its extensive antibiotic resistance via complex mechanisms, treatment options are very limited and the patients often have poor prognosis (Group of Infectious Diseases RDBoCMA, 2018). According to the China Bacterial Resistance Surveillance Network, less than 5% of CRAB were resistant to tigecycline and polymyxin, making regimen based on these antibiotics the primary treatment (Paul et al., 2022). Due to the high cost of polymyxin, tigecycline is more widely used in China.

Tigecycline is a minocycline derivative with antimicrobial activity against Gram-positive and Gram-negative bacteria, anaerobes and atypical pathogens. Its unique pharmacological mechanism provides good antibacterial activity against multidrug-resistant bacteria, include CRAB (Giammanco et al., 2017; Rodriguez-Bano et al., 2018). However, accumulating adverse event reports have raised safety concerns regarding tigecycline. Tigecycline may cause increased international normalized ratio (INR), prolonged activated partial thromboplastin time (APTT) and prolonged prothrombin time (PT) according to manufacturer’s label (Tygacil, 2013). Some studies suggest that tigecycline has a more pronounced effect on fibrinogen (FIB) decline (Liu et al., 2021; Leng et al., 2022; Yang et al., 2022).

Cefoperazone/sulbactam is an β-lactam/β-lactamase inhibitor combination, that is, effective for intra-abdominal, urinary tract, and respiratory infections. Sulbactam enhances the activity of cefoperazone and is used in cases of moderate-to-severe infection (Drawz and Bonomo, 2010), and is effective against ESBL-producing *E. coli* and *K. pneumoniae* (Su et al., 2018). Cefoperazone/sulbactam is widely used in China to treat hospital-acquired infection caused by CRAB; however, studies suggested that cefoperazone/sulbactam is associated with coagulation disorders (Greenberg et al., 1987; Xin et al., 2013). Case reports have demonstrated that patients receiving cefoperazone/sulbactam have experienced major bleeding, such as upper gastrointestinal bleeding, hematuria and abdominal wall hematoma (Wong et al., 2006; Cai et al., 2016). A possible mechanism for the coagulation abnormalities associated with cefoperazone may be its N-methylthio-tetrazole (NMTT) side chain, which induces vitamin K deficiency and leads to hypoprothrombinemia and bleeding (Lipsky, 1983; Mueller et al., 1987; Strom et al., 1999; Cai et al., 2016). Strom’s retrospective cohort study found that cefoperazone was linked to a higher risk of hypoprothrombinemia compared to the antibiotics without NMTT (Strom et al., 1999). A nested case-control study found that cefoperazone may increase the risk of bleeding compared to other antibiotics (Chen et al., 2016).

Patients with CRAB infection are usually critically ill, making appropriate antibiotic choice particularly important (Garnacho-Montero et al., 2015). As tigecycline is generally not used as a monotherapy due to heterogeneous resistance (Liu et al., 2019; Paul et al., 2022), cefoperazone/sulbactam plus tigecycline is the most used combination regimen for CRAB in China (Garnacho-Montero et al., 2015). However, both cefoperazone/sulbactam and tigecycline could increase the risk of bleeding. There is limited data as to whether the combination of these two agents increases the risk of bleeding compared to other tigecycline-based combination regimens. Therefore, we conducted this retrospective cohort and case-control study to investigate whether tigecycline plus cefoperazone/sulbactam increases the risk of bleeding compared with other tigecycline-based combination regimens, and investigate the risk factors associated with tigecycline-associated hypofibrinogenemia.

## Materials and methods

### Study design and patient selection

This was a multi-methods, multicenter, retrospective investigation of hospitalized patients receiving tigecycline-based combination therapy at three hospitals, including a cohort study and a case-control study. Each participating center (Shanxi Provincial Peoples Hospital, Shanxi Provincial Cancer Hospital and Yuncheng Central Hospital) was a tertiary hospital with over 2,200 inpatient beds. The study protocol was approved by the Ethics Committee of Shanxi Provincial People’s Hospital (2022-280). Researchers waived the need to obtain written informed consent due to its retrospective nature.

The enrolled population was divided into groups based on adjunct medication. The inclusion criteria were as follows: patients who received tigecycline-based treatment for at least 3 days, older than 18 years, high (loading dose 200 mg followed by 100 mg twice per day) or low tigecycline dose (loading dose 100 mg followed by 50 mg twice per day). The exclusion criteria were as follows: missing data; abnormal coagulation function before treatment; simultaneous use of drugs affecting coagulation, including aspirin, low molecular weight heparin, warfarin, clopidogrel, ticagrelol, and rivaroxaban; sepsis; liver failure; the number of cases in each group is less than 50.

The normal range of coagulation function was as follows in our hospital: APTT 25.1–36.5 s; PT 9.9–12.8 s; FIB 2.38–4.98 g/L. Hypofibrinogenemia was defined as plasma FIB <2.0 g/L (Leng et al., 2022). Naranjo score ≥4 was defined as tigecycline-associated hypofibrinogenemia in this study.

A total of 451 participants who met the inclusion/exclusion criteria between January 2018 and June 2022 at any of the three included hospitals were enrolled in this study.

### Cohort study

In this retrospective cohort study, we collected basic data for the enrolled patients and compared the baseline characteristics and coagulation data between patients treated with tigecycline plus cefoperazone/sulbactam, and patients treated with other tigecycline-based combination therapy.

### Case-control study

A case-control study was conducted to investigate the risk factors for tigecycline-associated hypofibrinogenemia in patients treated with tigecycline. The enrolled patients were divided into a hypofibrinogenemic and a non-hypofibrinogenemic group. We then assessed the relationship between tigecycline-associated hypofibrinogenemia and patient demographics, comorbidities, medication regimens and baseline laboratory tests.

## Data collection

We collected clinical information from the hospital information system including: age, sex, underlying diseases, site of infection, tigecycline dosage, duration of tigecycline treatment, concomitant drugs, PT, APTT, FIB, bleeding events, hemoglobin level, platelet count, transaminase level, albumin level, creatinine level. All laboratory tests performed from 48 h prior to tigecycline administration until 48 h after discontinuation of tigecycline were recorded.

### Statistical analysis

A mathematician not involved in the study procedures or patient assessment performed the statistical analyses using IBM-SPSS version 23.0. Shapiro-Wilk test was used to test normality. Statistical descriptions of quantitative variables are expressed as medians and interquartile range (IQR) or mean and standard deviations. Univariate comparisons were performed using the chi-square test for qualitative variables, and Student’s *t*-test or the Kruskal–Wallis H test for continuous variables, as appropriate. Mixed linear models were used to analyze the effects of different medication regimens and time factors on APTT, PT, and FIB levels. The Kruskal–Wallis H test was used for comparison between groups. The paired samples Wilcoxon test was used to compare the parameters before (T_1_) and after medication (T_2_). The risk factors for hypofibrinogenemia were analyzed by multivariate logistic regression. The receiver operating characteristic (ROC) curve was plotted using hypofibrinogenemia and course of treatment to determine the area under the curve (AUC) with 95% confidence interval. The ROC curve was used to determine the optimal cut-off point for course of treatment in all patients taking tigecycline.

## Results

The study cohort comprised 451 patients, divided into three groups: tigecycline plus cefoperazone/sulbactam (Group A), tigecycline plus carbapenems (Group B) and tigecycline plus β-lactam antibiotics without NMTT side chains (Group C). Group A contained 193, Group B contained 200, and Group C contained 58 patients, with no significant differences in age, sex, treatment duration, daily dose, occurrence of hypofibrinogenemia and bleeding events between the three groups (Table 1).

**TABLE 1:** Baseline information.

	Group A	Group B	Group C	*P*
Number of patients	193	200	58	
Sex (n, %)				
Male patients	154 (79.79)	146 (73.00)	44 (75.86)	0.285
Female patients	39 (20.21)	54 (27.00)	14 (24.14)	
Age, years *(P*_*25*_ *,P*_*75*_ *)*	65 (54,73)	65 (54,75)	63.5 (49.5,76)	0.748
Underlying diseases (n)				
Tumor	19	13	5	
Diabetes	6	19	6	
CKD	6	2	4	
CHF	0	3	1	
COPD	20	10	3	
Site of infection, n (%)				
Intra-abdominal	7 (3.63)	10 (5)	7 (12.1)	
Pneumonia	180 (93.26)	180 (9)	48 (82.8)	
SSTI	2 (1.04)	4 (2)	3 (5.2)	
Other	4 (2.07)	6 (3)	0	
ICU admission, n (%)	48 (24.87)	44 (22.00)	13 (22.41)	0.786
PCT	3.35 ± 13.07	4.39 ± 14.81	2.98 ± 6.47	0.972
Treatmentduration, days (*P*_*25*_ *,P*_*75*_)	7 (5,10.5)	7 (5,10)	7 (5,11)	0.369
Daily dose				
100 mg, n (%)	161 (83.4)	163 (81.5)	52 (89.7)	0.340
200 mg, n (%)	32 (16.6)	37 (18.5)	6 (10.3)	
Hypofibrinogenemia				
Yes, n (%)	75 (38.86)	93 (46.50)	23 (39.66)	0.281
No, n (%)	118 (61.14)	107 (53.50)	35 (60.34)	
Bleeding events, n (%)	19 (9.84)	19 (9.5)	7 (12.07)	0.845

Group A, tigecycline plus cefoperazone/sulbactam; Group B, tigecycline plus carbapenems; Group C, tigecycline plus β-lactam antibiotics without N-methylthio-tetrazole side chains; CKD, Chronic kidney disease; CHF, Congestive heart failure; COPD, Chronic obstructive pulmonary disease; SSTI, Skin and soft tissue infections.

The results of mixed linear models are shown in Table 2. The estimates between group B and group A in PT and APTT were 1.29 (*p* < 0.01) and 1.95 (*p* < 0.01) respectively; both PT and APTT were significantly longer in group B than in group A. While estimates between group C and group A in PT and APTT were −0.66 (*p* > 0.05) and 0.10 (*p* > 0.05) respectively, there was no significant difference. For both PT and APTT, the difference between T_1_ *versus* T_2_ was <0 (*p* < 0.05), suggesting PT and APTT were significantly prolonged after treatment for all patients.

**TABLE 2:** Results of mixed linear model.

Parameter	*p*	Estimates (95% CI)
PT Model		
Group B VS. Group A	<0.001^a^	1.29 (0.61–1.97)
Group C VS. Group A	0.199	−0.66 (−1.67–0.35)
T_1_ VS. T_2_	0.041^a^	−0.61 (−1.89∼-0.03)
interaction between Group B and T_1_ VS. other interaction	0.030^a^	−0.90 (−1.72∼-0.09)
interaction between Group C and T_1_ VS. other interaction	0.299	0.641 (−0.57–1.85)
APTT Model		
Group B VS. Group A	<0.012^a^	1.95 (0.42–3.48)
Group C VS. Group A	0.933	0.10 (−2.17–2.37)
T_1_ VS. T_2_	<0.001^a^	−2.93 (−4.32∼-1.55)
interaction between Group B and T_1_ VS. other interaction	0.127	−1.51 (−3.45–0.43)
interaction between Group C and T_1_ VS. other interaction	0.812	−0.35 (−3.23–2.53)
FIB Model		
Group B VS. Group A	0.105	−0.22 (−0.49–0.05)
Group C VS. Group A	0.539	−0.12 (−0.52–0.27)
T_1_ VS. T_2_	<0.001^a^	1.31 (1.02–1.61)
interaction between Group B and T_1_ VS. other interaction	0.642	0.09 (−0.31–0.52)
interaction between Group C and T_1_ VS. other interaction	0.540	−0.19 (−0.81–0.42)

a *p* < 0.05. PT, prolonged prothrombin time; APTT, activated partial thromboplastin time; FIB, fibrinogen; Group A, tigecycline plus cefoperazone/sulbactam; Group B, tigecycline plus carbapenems; Group C, tigecycline plus β-lactam antibiotics without N-methylthio-tetrazole side chains; T_1_, before treatment; T_2_, after treatment.

In FIB modelling, there was no significant difference in FIB levels between group B and A, or group C and A. But the estimated difference between T_1_ and T_2_ was 1.31 (*p* < 0.01), demonstrating that FIB levels significantly decreased after tigecycline-based combination therapy.

Since the interaction between group and time impacted PT level (*p* < 0.05), we further analyzed the influence of different time points and medication regimens on PT levels, and the change in PT level at different time points in different groups (Table 3). There were no significant differences in PT levels between the three groups before treatment (*p* = 0.14), but there were significant differences between the three groups (*p* < 0.01) after treatment. PT level in group B were significantly higher than that in groups A and C.

**TABLE 3:** Individual effect analysis of grouping and timing on PT levels.

	T_1_ (*P*_*25*_ *, P*_*75*_)	T_2_ (*P*_*25*_ *, P*_*75*_)	*p*
Group A	13.8 (12.6,15.4)	14.3 (12.9,16.4)^a^ ^*^	0.002^*^
Group B	14.4 (12.9,15.9)	15.2 (13.7,17.1)	<0.001^*^
Group C	13.7 (13.0,15.2)	13.9 (12.6,15.0)^b^ ^*^	0.524
*p*	0.140	<0.001^*^	

* *p* < 0.05.

Group A: tigecycline plus cefoperazone/sulbactam; Group B: tigecycline plus carbapenems; Group C: tigecycline plus β-lactam antibiotics without N-methylthio-tetrazole side chains; “a”: Group A compared to Group B; “b”: Group C compared to Group B; T_1_: before treatment; T_2_: after treatment.

Univariate logistic regression showed that the following variables were significant for the risk of hypofibrinogenemia at *p* < 0.05: gender, age, long treatment duration, high daily dose, total bilirubin, platelet, erythrocyte, low basal fibrinogen (Table 4). In the multivariate analysis, patients older than 80 years had a higher risk of hypofibrinogenemia compared with those under 50 (OR: 2.85, 95% CI: 1.07–7.60). Furthermore, long treatment duration (OR: 1.29, 95% CI: 1.19–1.41), high daily dose (OR: 2.6, 95% CI: 1.29–5.25), total bilirubin (OR: 1.01, 95% CI: 1.01–1.02) and low basal fibrinogen (OR: 1.32, 95% CI: 1.14–1.63) also were independent risk factors of hypofibrinogenemia.

**TABLE 4:** Risk factors for hypofibrinogenemia by logistic regression.

Risk factors	Univariate analysis		Multivariate analysis	
	Or (95% CI)	*p*	Or (95% CI)	*p*
Gender (Female VS. Male)	1.59 (1.03–2.47)	0.036^a^	1.34 (0.66–2.71)	0.417
Age, years				
≥50 VS. <50	0.90 (0.47–1.72)	0.748	1.13 (0.44–2.89)	0.794
≥60 VS. <50	1.54 (0.87–2.74)	0.141	1.99 (0.83–4.75)	0.124
≥70 VS. <50	1.98 (1.07–3.69)	0.031^a^	2.03 (0.76–5.39)	0.156
≥80 VS. <50	2.74 (1.39–5.37)	0.003^a^	2.85 (1.07–7.60)	0.036^a^
Treatment duration	1.14 (1.08–1.19)	<0.001^a^	1.29 (1.19–1.41)	<0.001^a^
Daily dose (200 mg VS. 100 mg)	1.55 (0.94–2.55)	0.085	2.60 (1.29–5.25)	0.008^a^
Total bilirubin	1.01 (1.01–1.02)	0.020^a^	1.01 (1.01–1.02)	0.003^a^
ALT	0.99 (0.99–1.01)	0.124		
AST	1.00 (0.99–1.01)	0.702		
Serum creatinine	1.00 (1.00–1.01)	0.139		
White blood cell	1.02 (0.99–1.05)	0.191		
Erythrocyte	0.99 (0.98–0.99)	0.022^a^	0.99 (0.97–1.00)	0.082
Platelet	0.99 (0.98–0.99)	0.008^a^	0.99 (0.99–1.00)	0.052
Basal fibrinogen	1.45 (1.18–1.86)	0.005^a^	1.32 (1.14–1.63)	0.023^a^

a *p* < 0.05.

ALT, alanine aminotransferase; AST, aspartate aminotransferase.

The ROC curve was used to evaluate the relationship between tigecycline treatment duration and tigecycline-related hypofibrinogenemia. AUC of treatment duration for the high dose was 0.70 (predictive ability of *p* < 0.05), and for the low dose was 0.73 (*p* < 0.05). Treatment duration ≥6 days for high-dose regimen and treatment duration ≥11 days for low-dose regimen were selected as the cutoff points for predicting hypofibrinogenemia during tigecycline treatment (Table 5).

**TABLE 5:** Results of ROC.

Variable	AUC	Sensitivity	Specificity	Cut-off (days)
Treatment duration-High dose	0.70	0.87	0.46	6.00
Treatment duration-Low dose	0.73	0.64	0.69	11.00

## Discussion

Previous reports suggested that both tigecycline and cefoperazone sulbactam can cause coagulopathies (Wong et al., 2006; Rossitto et al., 2014; Yilmaz Duran et al., 2018; Wang et al., 2020; Liu et al., 2021; Leng et al., 2022). The widespread use of tigecycline in combination with cefoperazone/sulbactam has increased physicians’ concerns about an increased risk of bleeding associated with this regime. We therefore conducted a multicenter, retrospective, controlled study to assess the coagulopathies associated with tigecycline. To the best of our knowledge, this is the first study to compare the coagulation disorders between tigecycline plus cefoperazone/sulbactam and other tigecycline-based combination therapies, this is also the largest study of tigecycline-induced coagulopathy. In our cohort study, we evaluated the effect of tigecycline with carbapenems, or tigecycline with a β-lactam on coagulation when compared to tigecycline with cefoperazone/sulbactam as a control, given that tigecycline plus cefoperazone/sulbactam is the primary regimen for the treatment of CRAB infections in China.

The results of the mixed linear model showed that APTT and PT were significantly prolonged, and FIB decreased in all enrolled patients after receiving tigecycline-based therapy. It is generally reported that carbapenems have little effect on coagulation (Leng et al., 2022). While among β-lactam antibiotics, only NMTT-side-chain-containing antibiotics (such as cefoperazone and cefmetazole) are known to cause coagulopathies (Chen et al., 2016). The coagulation dysfunction induced by tigecycline has been confirmed by various studies (Rossitto et al., 2014; Liu et al., 2021; Leng et al., 2022). Therefore, we believe that the coagulopathies observed in the patients in this study are caused by tigecycline. However, the underlying mechanism for tigecycline-induced coagulopathies remains unclear.

The known mechanism of antibiotic-associated coagulopathy is through reduced intestinal flora or inhibited vitamin K_2_ synthesis; however these mechanisms do not influence the production of fibrinogen (Haden, 1957; Shirakawa et al., 1990; Conly and Stein, 1992). The patients did not have symptoms suggestive of overconsumption, such as disseminated intra-vascular coagulation, primary active bleeding, and clotting factors degradation accelerated by acidosis. We therefore concluded that the reduction of FIB has no obvious relationship with clotting factors consumption (Martini, 2009). Tigecycline-induced cytokine inhibition may be responsible for reduced fibrinogen production. Vasse et al. (1996) demonstrated that tigecycline can inhibit IL-6 expression in leucocytes; since IL-6 increases plasma fibrinogen by stimulating gene expression, tigecycline may lead to a decrease in plasma fibrinogen by inhibiting IL-6 synthesis (Fuller et al., 1985).

Both PT and APTT were significantly higher in group B than A, contrary to our initial assumptions. We hypothesize this is due to tigecycline’s greater effect on coagulation than cefoperazone/sulbactam. The proportion of patients receiving high-dose tigecycline in group B (18.5%) was higher than in groups A (16.6%) and C (10.3%); tigecycline induced coagulopathy is usually dose dependent (Cui et al., 2019). PT levels in group B were significantly higher than in groups A and C after medication (Table 3), which supports our hypothesis. There were no differences in APTT and PT between group C and A. Given that carbapenems and β-lactam antibiotics without NMTT side chains had little effect on coagulopathy, and there was no significant difference in bleeding events between the three groups, we did not consider tigecycline with cefoperazone/sulbactam to have an increased risk of bleeding when compared to tigecycline with carbapenem, or tigecycline with a β-lactam. Leng’s retrospective research suggested tigecycline plus cefoperazone/sulbactam did not increase the risk of coagulopathy compared to tigecycline monotherapy, which is consistent with our study (Leng et al., 2019).

Multivariate logistic regression showed that patients >80 years, long treatment duration, high daily dose, high total bilirubin and low basal fibrinogen were independent risk factors for tigecycline-associated hypofibrinogenemia. However, data on the correlation between age and hypofibrinogenemia is inconclusive. Research from China and Austria found that the incidence of hypofibrinogenemia and age are unrelated (Zhang et al., 2015; Campany-Herrero et al., 2020; Hu et al., 2020; Xia and Jiang, 2020; Treml et al., 2021; Leng et al., 2022). However, our study, alongside a few others, suggests that advanced age is a risk factor for tigecycline-associated hypofibrinogenemia (Zhang et al., 2020; Liu et al., 2021). Fibrinogen is synthesized in the liver; we speculate that the hepatic synthetic ability declines in patients with advanced age, which is supported by our finding that total bilirubin levels are also associated with hypofibrinogenemia. Hypofibrinogenemia can be caused by liver disease due to reduced liver synthetic function or dysfibrinogenemia, we suggest this is the reason why hypofibrinogenemia is associated with total bilirubin (May et al., 2021).

Our study showed that a dose of tigecycline higher than 100 mg/day is a risk factor for hypofibrinogenemia, which is consistent previous studies (Campany-Herrero et al., 2020; Xia and Jiang, 2020; Treml et al., 2021). As tigecycline-induced coagulopathy is usually dose dependent, this may explain why hypofibrinogenemia is more likely to occur in the high-dose group.

Long term tigecycline treatment is associated with hypofibrinogenemia (Campany-Herrero et al., 2020; Xia and Jiang, 2020; Liu et al., 2021). Coagulopathy developed at a median of 6 days after tigecycline treatment in one study, which is similar to our research (Hu et al., 2020). However, there are two regimens of tigecycline, high-dose and low-dose, commonly used in clinical practice. Further analysis showed that the cut-off for hypofibrinogenemia in high-dose patients was 6 days, and 11 days for low-dose patients; far below other findings of 4 weeks (Campany-Herrero et al., 2020). However, our study involved a larger cohort and stricter inclusion criteria. In addition, this study was conducted in a Chinese population, and the previous study conducted in a Spanish population. Therefore, it is necessary to closely monitor FIB level when receiving high-dose tigecycline treatment.

Our study has several limitations. First, this is a retrospective observational study, and there may be bias and confounders that we did not control for. Second, Yang et al. (2022) reported serum concentration as a predictor of tigecycline-induced hypofibrinogenemia, but we were unable to include therapeutic drug monitoring for tigecycline. Third, the association between mortality and treatment regimen was not investigate. Despite these limitations, we investigated the effects of tigecycline with cefoperazone/sulbactam on coagulation function, and risk factors for tigecycline-associated hypofibrinogenaemia, which will facilitate further research in this field.

## Conclusion

In conclusion, tigecycline with cefoperazone/sulbactam did not increase the risk of bleeding compared to tigecycline with carbapenem, or tigecycline with β-lactam antibiotics without NMTT-side-chains. Patients older than 80 years, on long-term tigecycline treatment, high daily dose, high total bilirubin and low basal fibrinogen were independent risk factors for tigecycline-associated hypofibrinogenemia. Coagulation function should be closely monitored in patients receiving tigecycline treatment.

## Data Availability

The original contributions presented in the study are included in the article/Supplementary Material, further inquiries can be directed to the corresponding author.

## Abbreviations

AUC, area under the curve; APTT, activated partial thromboplastin time; CRAB, carbapenem-resistant *Acinetobacter* baumannii; IQR, interquartile range; PT, prothrombin time; FIB, fibrinogen; NMTT, N-methylthio-tetrazole; ROC, receiver operating characteristic; T1, before medication; T2, after medication.
