# Erratum: Comparison of bleeding risk and hypofibrinogenemia-associated risk factors between tigecycline with cefoperazone/sulbactam therapy and other tigecycline-based combination therapies

**DOI:** 10.3389/fphar.2024.1365927

**Published:** 2024-01-15

**Authors:** Frontiers Production Office

**Affiliations:** Frontiers Media SA, Lausanne, Switzerland

**Keywords:** tigecycline, cefoperazone/sulbactam, carbapenems, β-lactam antibiotics, coagulation disorders, hypofibrinogenaemia

Due to a production error, there was a mistake in Table 1 as published. The table erroneously contained references. The corrected Table 1 appears below.

**TABLE 1:** Baseline information.

	Group A	Group B	Group C	*P*
Number of patients	193	200	58	
Sex (n, %)				
Male patients	154 (79.79)	146 (73.00)	44 (75.86)	0.285
Female patients	39 (20.21)	54 (27.00)	14 (24.14)	
Age, years *(P*_*25*_ *,P*_*75*_ *)*	65 (54,73)	65 (54,75)	63.5 (49.5,76)	0.748
Underlying diseases (n)				
Tumor	19	13	5	
Diabetes	6	19	6	
CKD	6	2	4	
CHF	0	3	1	
COPD	20	10	3	
Site of infection, n (%)				
Intra-abdominal	7 (3.63)	10 (5)	7 (12.1)	
Pneumonia	180 (93.26)	180 (9)	48 (82.8)	
SSTI	2 (1.04)	4 (2)	3 (5.2)	
Other	4 (2.07)	6 (3)	0	
ICU admission, n (%)	48 (24.87)	44 (22.00)	13 (22.41)	0.786
PCT	3.35 ± 13.07	4.39 ± 14.81	2.98 ± 6.47	0.972
Treatmentduration, days (*P*_*25*_ *,P*_*75*_)	7 (5,10.5)	7 (5,10)	7 (5,11)	0.369
Daily dose				
100 mg, n (%)	161 (83.4)	163 (81.5)	52 (89.7)	0.340
200 mg, n (%)	32 (16.6)	37 (18.5)	6 (10.3)	
Hypofibrinogenemia				
Yes, n (%)	75 (38.86)	93 (46.50)	23 (39.66)	0.281
No, n (%)	118 (61.14)	107 (53.50)	35 (60.34)	
Bleeding events, n (%)	19 (9.84)	19 (9.5)	7 (12.07)	0.845

Group A, tigecycline plus cefoperazone/sulbactam; Group B, tigecycline plus carbapenems; Group C, tigecycline plus β-lactam antibiotics without N-methylthio-tetrazole side chains; CKD, Chronic kidney disease; CHF, Congestive heart failure; COPD, Chronic obstructive pulmonary disease; SSTI, Skin and soft tissue infections.

The publisher apologizes for this mistake. The original version of this article has been updated.

